# Loss of *Twist1* leads to disruption of ciliary length, endocytic vesicle dynamics, and cell–cell junctions during neural tube formation

**DOI:** 10.1002/dvdy.70109

**Published:** 2026-01-06

**Authors:** Derrick Thomas, Brittany M. Hufft-Martinez, Zarna Lalwani, Vi Pham, Mary Elmeniawi, An J. Tran, Jianming Xu, Irfan Saadi, Walid D. Fakhouri

**Affiliations:** 1 Department of Diagnostic and Biomedical Sciences, Center for Craniofacial Research, School of Dentistry, University of Texas Health Science Center at Houston, Houston, Texas, USA; 2 Department of Cell Biology and Physiology, The University of Kansas Medical Center, Kansas City, Kansas, USA; 3 Department of Molecular and Cellular Biology, Baylor College of Medicine, Houston, Texas, USA; 4 Department of Pediatrics, McGovern Medical School, University of Texas Health Science Center at Houston, Houston, Texas, USA

**Keywords:** cell shape changes, cell–cell junction, endosomal recycling, neural fold bending, neuroectoderm

## Abstract

**Background:**

Endocytosis constitutes a fundamental cellular process governing development through coordinated regulation of plasma membrane remodeling and ciliogenesis, processes essential for cell shape changes and tissue development. Although *Twist1* null embryos display complete cranial neural tube (NT) closure defects and conditional knockout in neuroectoderm disrupts cranial neural crest cell fate determination and delamination, the function of TWIST1 in NT morphogenesis remains unknown. We investigated the basis underlying neuroectodermal morphological abnormalities in TWIST1 mutant embryos, specifically the formation of ectopic lateral bending points and cellular disorganization, by examining *Twist1’s* role in cilia formation, adherens junction integrity, and endocytic vesicle dynamics.

**Results:**

Immunofluorescence analysis revealed that cytosolic TWIST1 colocalizes with β-catenin and endocytic regulators LRP2 and RAB11B along the apical surface of cranial neuroectoderm. *Twist1* knockout resulted in reduced ciliary length and number. Quantitative polymerase chain reaction (PCR) and Western blot analyses demonstrated upregulation of RAB11B and β-catenin at mRNA and protein levels in *Twist1* mutants. This molecular dysregulation coincided with increased accumulation of apical endocytic vesicles and altered expression profiles of endocytic component genes, ultimately modifying the apical neuroectodermal cell–cell junctions.

**Conclusion:**

Our findings establish TWIST1 as a crucial factor for neuroectodermal morphology, demonstrating its importance in ciliogenesis, endocytic vesicle dynamics, and cell–cell integrity.

## 1 | INTRODUCTION

Craniofacial disorders are the second most common congenital birth defects worldwide, arising from disruptions in neural tube (NT) formation and cranial neural crest cell (CNCC) development.^1^ The morphogenesis of NT and CNCC depends on complex and dynamic cellular and tissue rearrangements orchestrated by key morphogenetic processes, including cell proliferation, apical constriction, cell shape changes, cell intercalation, fate transition, and delamination, all critical for subsequent craniofacial development.^2,3^ Most frontal craniofacial bone and cartilage structures are derived from CNCCs specified in the neural plate borders of the neuroectoderm. These specified cells undergo epithelial-to-mesenchymal transition (EMT) and delamination from neural fold edges to become multipotent migratory mesenchymal cells that reach the frontonasal process and pharyngeal arches where they differentiate into bone, cartilage, neurons, and smooth muscle.^4–7^ While numerous contributory genes and signaling pathways regulating NT closure and CNCC formation have been identified, mechanistic understanding of the cellular and molecular modifications during cell fate transition and NT closure remains poorly understood.

TWIST1 is a basic helix–loop–helix transcription factor that serves as a master regulator of EMT in various cancer cells, promoting metastasis and often contributing to chemoresistance.^8–10^ During embryogenesis, *Twist1* regulates developmental genes involved in mesoderm differentiation, mesenchymal cell survival, osteogenic differentiation, and numerous cancer-associated pathways.^8^ Previous studies demonstrated that NT closure failure in *Twist1* null mice results from reduced proliferative capacity of adjacent mesodermal cells, which is critical for proper neural fold elevation and potentially NT closure.^11^ However, recent studies have demonstrated that *Twist1* mRNA and protein are expressed in neuroectoderm during NT development, detected by scRNA-seq and immunofluorescent staining,^12–14^ findings validated by quantitative in situ mRNA hybridization (Stereo-seq), suggesting a direct role in NT development and closure.^13^

Mutations in the human *TWIST1* gene can cause multiple common birth defects, including craniosynostosis, hypertelorism, ptosis, cleft palate, and limb abnormalities, while haploinsufficiency leads to Saethre–Chotzen syndrome.^15–21^ Notably, many of these disorders overlap with features observed in ciliopathies, genetic defects caused by dysfunction of the primary cilia, which present with craniofacial anomalies in approximately 30% of cases.^22,23^ Similarly, loss of function and knockout of *Twist1* in mice produce several overlapping phenotypes with human disorders, including cleft palate, craniosynostosis, and limb abnormalities.^12,18,24,25^ We previously demonstrated that *Twist1* cko in CNCCs and *Twist1* phospho-deficient mutant mouse embryos exhibit cranial NT clefts and craniofacial bone loss.^12^ We showed that TWIST1 is necessary for suppressing adherens junction proteins and epithelial markers during the EMT of pre-migratory CNCCs as they emerge from neural plate borders.^12^ These *Twist1* mutant mouse models confirm the critical function of TWIST1 in regulating NT formation.

Notably, TWIST1 expression overlaps with β-catenin at the apical surface of neuroectodermal cells and colocalizes with endocytic factors LRP2 and RAB11B in neuroectodermal cells.^14^ Furthermore, the cytoplasmic TWIST1 protein interacts with Tight Junction Protein 1 (TJP1) in cancer cells, and with δ-catenin in neuroectoderm and prostate cancer cells to stabilize cytoplasmic TWIST1 by ubiquitin modification.^26–28^ TWIST1 also functions as a mechano-mediator for matrix stiffness, which promotes nuclear translocation of TWIST1 by releasing it from the cytoplasmic binding partner Ras GTPase-activating protein-binding protein 1.^26^ These findings propose a new function of cytoplasmic TWIST1 through interactions with the β/δ-catenins and TJP1 to stabilize the protein and potentially modulate membrane-associated proteins before nuclear translocation. Therefore, the function of cytosolic TWIST1 and the importance of its interaction with β/δ-catenins require further molecular and cellular investigation during development and in cancer diseases.^12^

Endosomes are critical organelles formed by the cell membrane and Golgi apparatus, serving as vital regulators of signaling molecule internalization, translocation, ciliogenesis, and recycling of membrane-associated proteins.^29–32^ Among numerous genes associated with apical endosomal formation, *Lrp2* and *Rab11b* represent well-studied examples in neuroectodermal cells. LRP2, a transmembrane receptor, plays a crucial role in NT closure. Endosomal proteins RAB11B and VANGL2 recycle the cell membrane-associated proteins within the apical surface of the neural folds to decrease apical surface area, enabling proper neuroectodermal closure.^14^ LRP2 and RAB11B are highly expressed along the apical surface with elevated expression at the dorsal midline and lateral edges.^14^ Furthermore, endocytic trafficking proteins, such as RAB11B and RAB5 GTPase, regulate important aspects of ciliary membrane delivery and turnover. Additionally, RAB proteins, including RAB34, have been implicated in modulating cilia length and function. Together, RAB GTPases play a critical role in coordinating vesicular trafficking, membrane delivery, and signaling events required for the assembly and maintenance of the primary cilium.^33–36^

This study investigates *Twist1’s* impact on endosomal recycling proteins, ciliogenesis, and cell–cell junctions in neuroectodermal cells during NT closure and pre-migratory CNCC formation. We examined TWIST1 colocalization with endocytic vesicle markers, quantified mRNA and protein levels of membrane recycling proteins in *Twist1* null and conditional knockout mice, counted endocytic vesicles in *Twist1* mutant embryos, and performed quantitative analysis of cell–cell junctions to explain morphological and cellular changes in apical neuroectodermal cells. This study proposes a new role of TWIST1 in affecting primary ciliary length, endocytic vesicle dynamics, and cell–cell junctions in neuroectoderm during neural fold closure and CNCC formation.

## 2 | RESULTS

### 2.1 | Whole mount images and histological analysis of NT abnormalities

**FIGURE 1:**
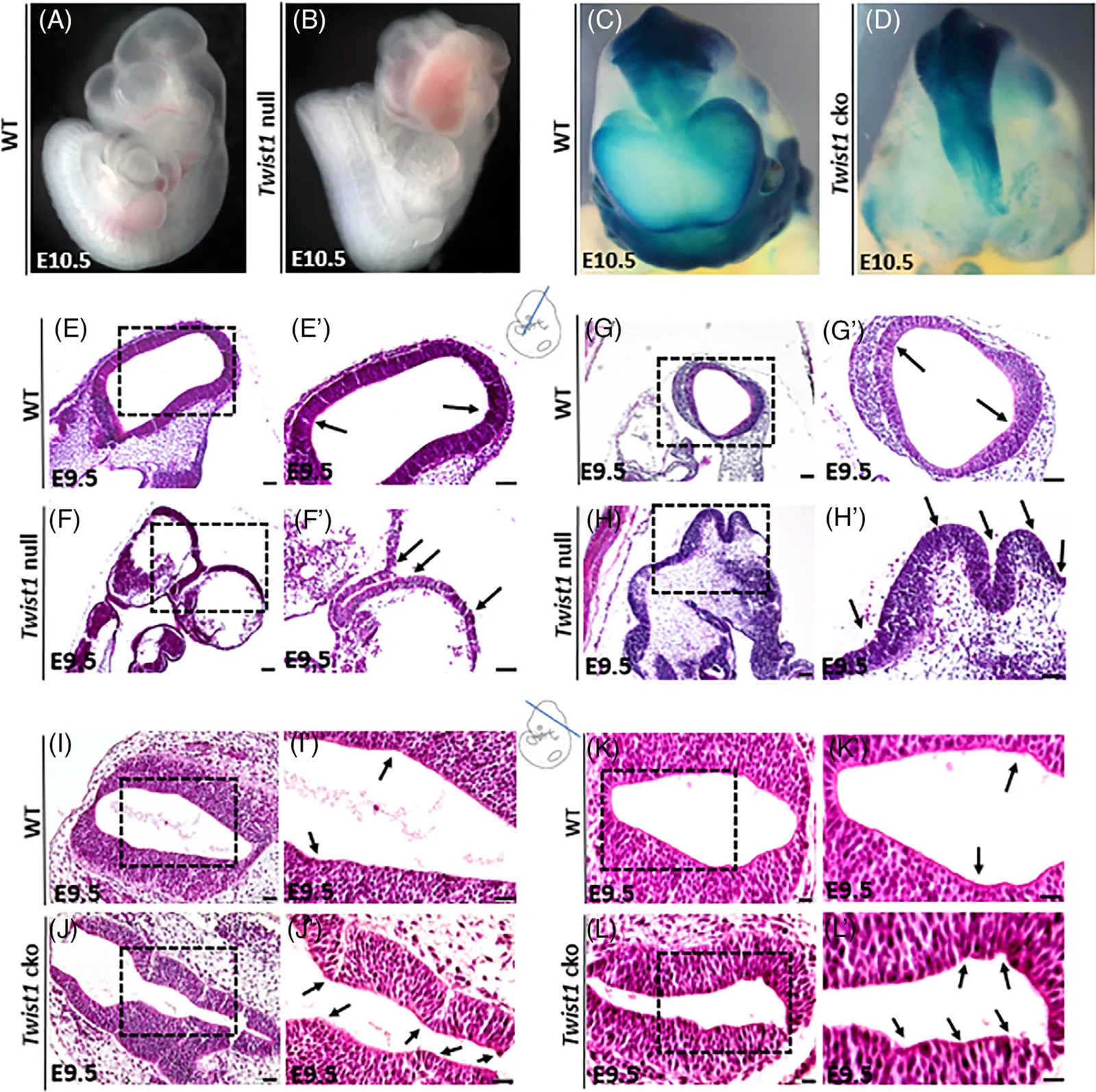
Whole mount embryos and histological analysis of neural fold development in *Twist1* mutant embryos. (A) Wild-type embryo at E10.5 showing a normal development and closure of neural tube. (B) *Twist1* null embryo exhibiting complete neural tube closure defects at E10.5. (C) Wild-type embryo at E10.5 showing a normal development and closure of neural tube (CNCC TM1). (D) *Twist1* cko embryo at E10.5 presents neural tube developmental defects and improper closure. (E, E′) H&E staining of E9.5 wild-type mouse embryo showing the morphology and closure of midbrain neural tube (NT) with organized bending points (black arrows) of neuroectodermal brain cortex. (F, F′) H&E staining of E9.5 *Twist1* null mouse exhibiting abnormal morphology and disorganized folding of the neuroectodermal folds. (G,G′) H&E staining of E9.5 wild-type mouse showing the morphology and closure of the hindbrain NT. (H, H′) H&E staining of E9.5 *Twist1* null mouse showing the severe abnormal morphology and disorganized folding of the mid- and hindbrain of neuroectodermal folds. (I, I′) H&E staining of E9.5 wild-type mouse showing proper morphology and closure of the hindbrain NT. (J, J’) H&E staining of E9.5 *Twist1* cko mouse showing the abnormal morphology of the neural folds with multiple bending points and expansion of neuroectoderm (black arrows). (K, K′) H&E staining of E9.5 wild-type mouse showing proper morphology and closure of the midbrain NT. (L, L’) H&E staining of E9.5 *Twist1* cko mouse showing the abnormal morphology of the neural folds with multiple bending points of neuroectoderm (black arrows).

A whole mount image of *Twist1* null embryo exhibits complete NT closure defects with multiple ectopic bending points, and *Twist1* cko embryo presents NT developmental defects and improper closure compared to wild-type (WT) littermates at E10.5 (Figure 1A–D). To further analyze morphological changes, we performed H&E staining on sections of mouse neural folds to show proper bending at the ventral midline and at the lateral dorsal regions important for NT closure (Figure 1E,E′,G, G′) while the *Twist1* null embryos exhibited multiple folding and elevation at the middle-lateral regions without the formation of proper bending points leading to complete failure of NT closure (Figure 1F,F′,H,H′). Similarly, control embryos showed proper folding and bending at the lateral dorsal regions (Figure 1I,I′,K,K′). The *Twist1* cko embryos showed narrow ventral midline bending and multiple lateral dorsal bending points. Also, the expansion of the neural folds varied from the dorsal to the midline regions (Figure 1J,J′,L,L′).

### 2.2 | TWIST1 expression in neural folds

**FIGURE 2:**
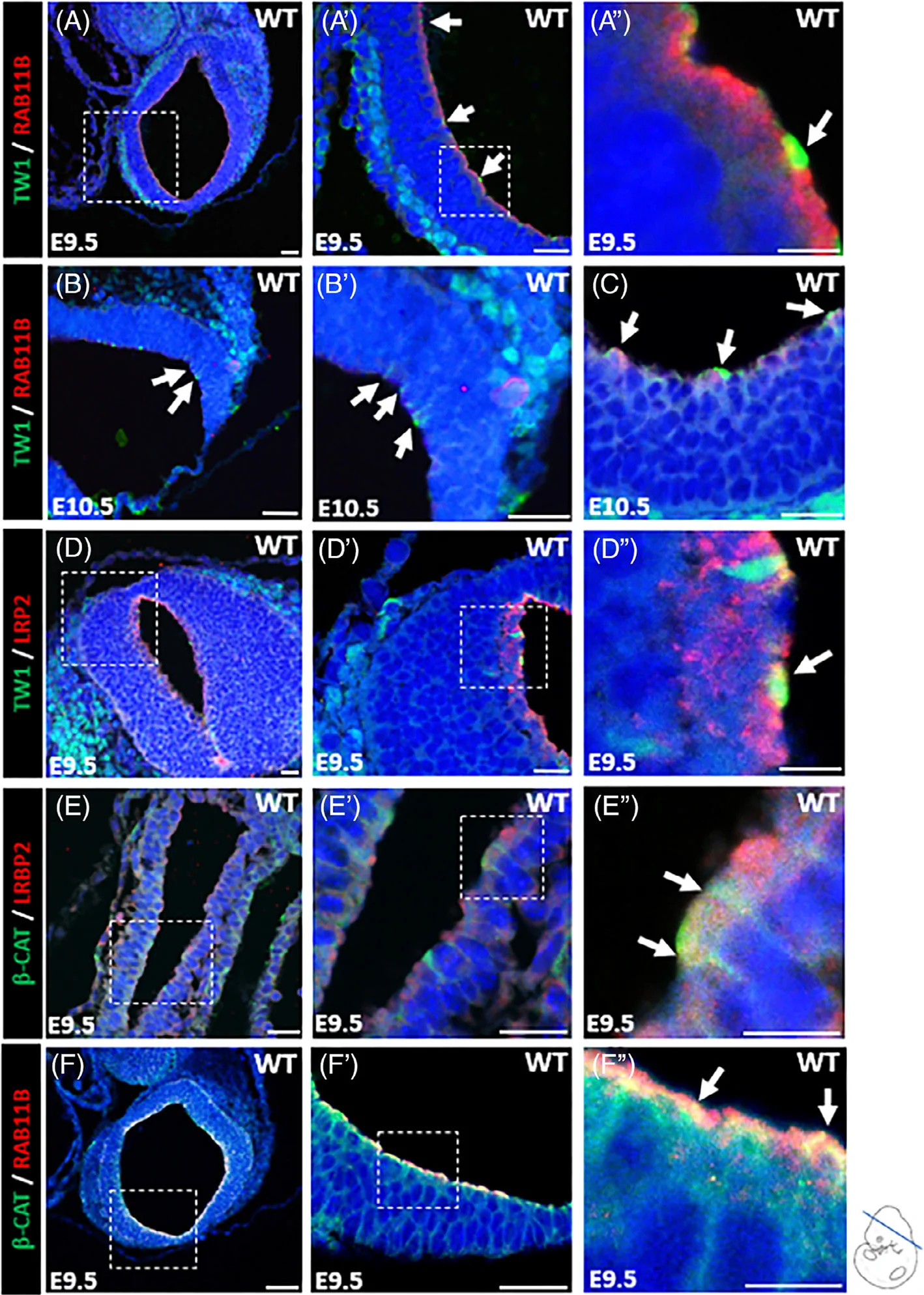
Expression pattern of TWIST1, endocytic vesicle markers, and adherens junction protein. (A, A″) Immunostaining of E9.5 wild-type mouse embryo showing TWIST1 protein expression in green at apical surface of neuroectodermal cells, and its expression overlaps with endocytic vesicle marker, RAB11B, in yellow (white arrow). TWIST1 nuclear expression was detected in the delaminated cranial neural crest cells along the neural tube (NT). (B, B′, C) Immunostaining of E10.5 wild-type mouse embryo showing TWIST1 protein expression in green at apical surface of neuroectodermal cells, and its expression overlaps with endocytic vesicle marker, RAB11B, in yellow (white arrow). TWIST1 nuclear expression was detected in the delaminated cranial neural crest cells along the NT and near bending sites. (D, D″): Immunostaining of E9.5 wild-type mouse embryo showing TWIST1 protein expression in green at apical surface of neuroectodermal cells, and its expression overlaps with endocytic vesicle marker, LRP2, in yellow (white arrow). (E, E″) Immunostaining of E9.5 wild-type mouse embryo showing the expression of β-catenin in green at apical-lateral side of neuroectodermal cells, and its expression overlaps with endocytic vesicle marker, LRP2, in yellow (white arrow). (F, F″) Immunostaining of E9.5 wild-type mouse embryo showing the expression of β-catenin in green at apical-lateral side of neuroectodermal cells, and its expression overlaps with endocytic vesicle marker, RAB11B, in yellow (white arrow).

Dual immunofluorescence (IF) staining of TWIST1/RAB11B, TWIST1/LRP2, RAB11B/β-catenin, and LRP2/β-catenin demonstrated that TWIST1 is expressed at the apical region of WT E9.5 and E10.5 mouse embryos’ dorsal neuroectodermal cells, and its expression overlaps with the apical endocytic markers RAB11B and LRP2 as indicated by the yellow fluorescent color (Figure 2A–D″). We also detected a strong nuclear expression of TWIST1 in the delaminated CNCCs along the neural folds and near the bending sites (Figure 2A,B,D). The expression of adherens junction protein β-catenin also overlaps at the dorsal lateral region with the endocytic vesicle marker LRP2 (Figure 2E,E″), and RAB11B (Figure 2F,F″) at the apical side of the neuroectodermal cells.

### 2.3 | Shortened cilia in *Twist1*^−/−^ neuroectoderm

**FIGURE 3:**
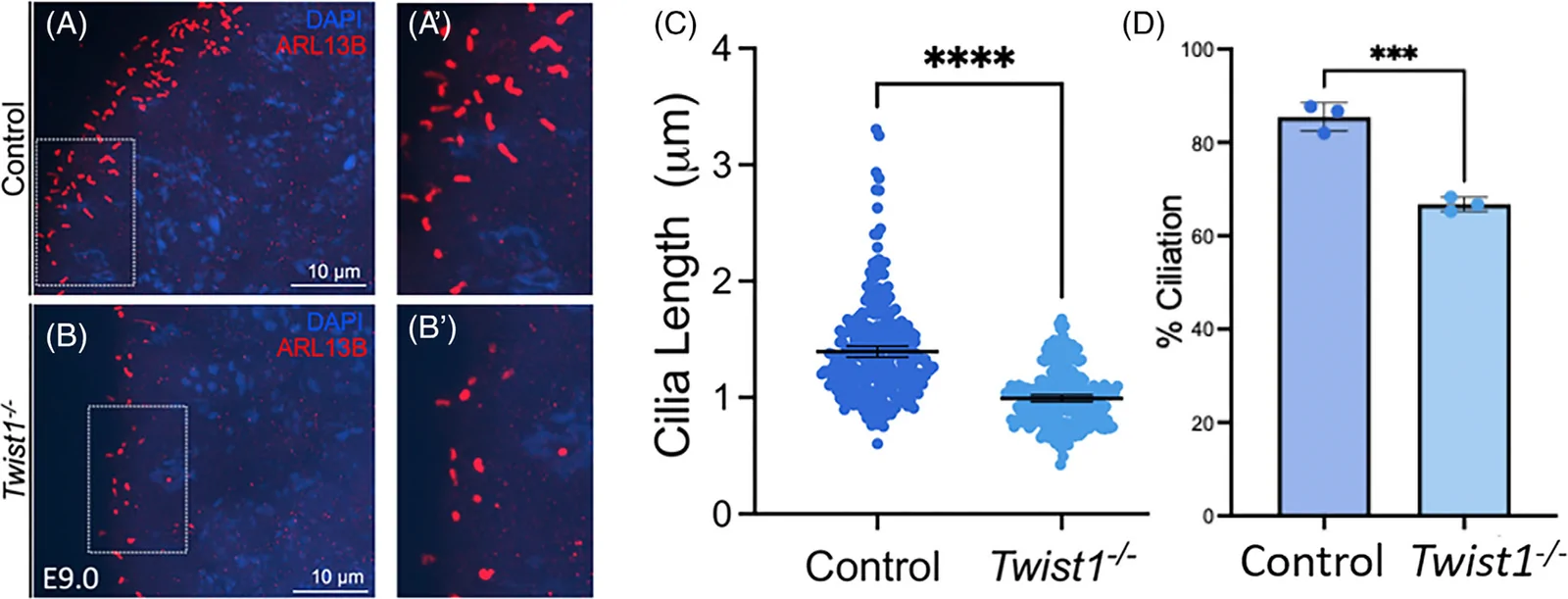
Primary cilia length is decreased in *Twist1*^−/−^ neuroectoderm. Immunostaining with ARL13B of control (A) and *Twist1*^−/−^ (B) mouse neuroectoderm from E9.0 embryos. (A’, B′): Magnification of boxed regions highlighting primary cilia. (C) Quantification of cilia length (μm) from E9.0–9.5 embryonic neuroectoderms stained with ARL13B. Approximately 50 primary cilia per embryo were measured from 6 control (*n* = 300) and 5 *Twist1*^−/−^ (*n* = 239) samples. (D) Quantification of percent ciliation from E9.0 to E9.5 embryonic neuroectoderms stained with ARL13B and DAPI. For percent ciliation quantification, 3 control (*n* = 530) and *Twist1*^−/−^ (*n* = 734) samples were measured.

We investigated primary cilia length using ARL13B, a ciliary membrane marker, to determine *Twist1’s* impact on ciliary length utilizing control and *Twist1*^−/−^ tissue from E9.0 and 9.5 embryos (Figure 3A–B′). We measured primary cilia length and found a significant reduction in *Twist1*^−/−^ cilia length compared to control (Figure 3C). We next looked at the ciliation in *Twist1*^−/−^ tissue from E9.0 and E9.5 embryos and observed a significant decrease in the number of ciliated cells compared to WT littermates (Figure 3D). This decrease in *Twist1*^−/−^ cilia length and percentage of ciliated cells suggest TWIST1 expression is necessary for normal ciliogenesis in the neuroectoderm.

### 2.4 | Quantification of mRNA and protein of endocytic and adhesion junction markers in *Twist1* null and cko

**FIGURE 4:**
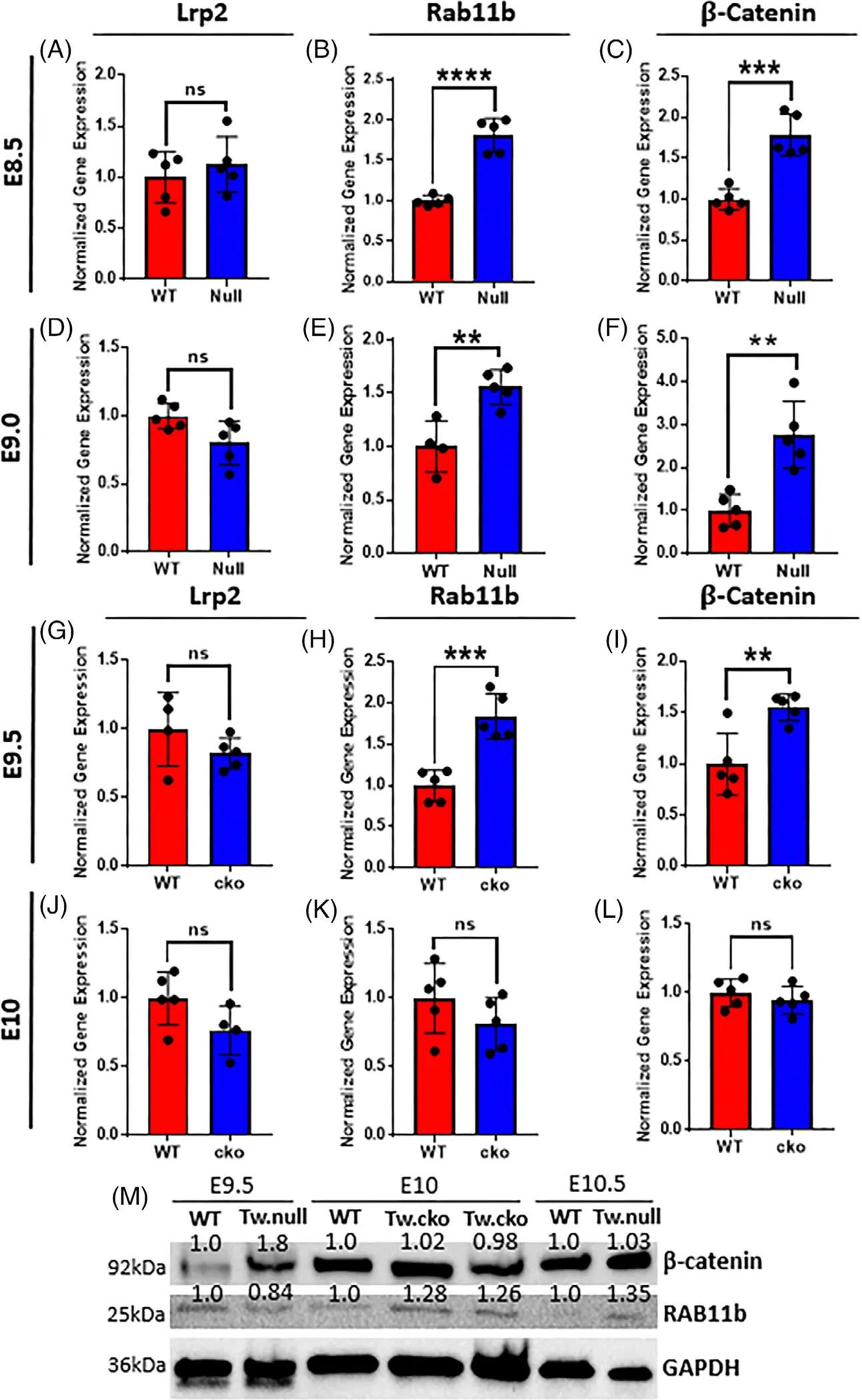
Quantitative analysis of endocytic vesicle markers and β-catenin in *Twist1* mutant embryos. (A–F) mRNA expression of *Lrp2*, *Rab11b*, and β-*catenin* in hindbrain of *Twist1* null embryos at E8.5 and E9. The expression of *Rab11b* and β-*catenin* significantly increased while no change in *Lrp2* was detected. (G–L) mRNA expression of *Lrp2*, *Rab11b*, and β-*catenin* in hindbrain of *Twist1* cko embryos at E9.5 and E10. The expression of *Rab11b* and β-*catenin* significantly increased at E9.5 while no significant change was detected in the expression of *Lrp2*, *Rab11b*, and β-*catenin* at E10 between mutant and wild-type mouse embryos. For statistical analysis, the * represents a *p*-value <.05, ** represents a *p*-value <.01, *** represents a *p*-value <.001, and ****represents a *p*-value <.0001. (M): Protein level of β-catenin and RAB11B was quantified in *Twist1* null and cko compared to corresponding wild-type (WT) embryos and the protein level was normalized to the housekeeping gene GAPDH. A slight increase in the protein level of both *Twist1* null and cko was detected compared to WT for β-catenin and RAB11B.

We observed no significant changes in LRP2 mRNA transcripts in *Twist1*^−/−^ and *Twist1*^*cko/−*^ compared to WT at E8.5, E9.0, E9.5, and E10, whereas an almost twofold increase in expression of RAB11B and β-catenin was detected at E8.5, E9.0, and E9.5 embryonic stages (Figure 4A–I). However, no significant changes in expression levels of RAB11B or β-catenin were observed for *Twist1*^*cko/−*^ at E10.0 (Figure 4J–L).

To evaluate the changes at the protein level, a Western blot was performed for total proteins extracted from *Twist1*^−/−^ and *Twist1*^*cko/−*^ samples and compared to the corresponding WT littermates. At E9.5, total β-catenin protein was increased in *Twist1*^−/−^ when compared to WT. A similar trend was observed at embryonic stage E10 and E10.5 in *Twist1*^−/−^ and *Twist1*^*cko/−*^, the level of β-catenin and RAB11B was elevated compared to WT. This shows that both β-catenin and RAB11B are expressed in earlier embryonic time points and their expression elevated in *Twist1* mutant embryos. We used GAPDH as a loading control and for normalization (Figure 4M).

### 2.5 | Immunofluorescent staining of endocytic markers

**FIGURE 5:**
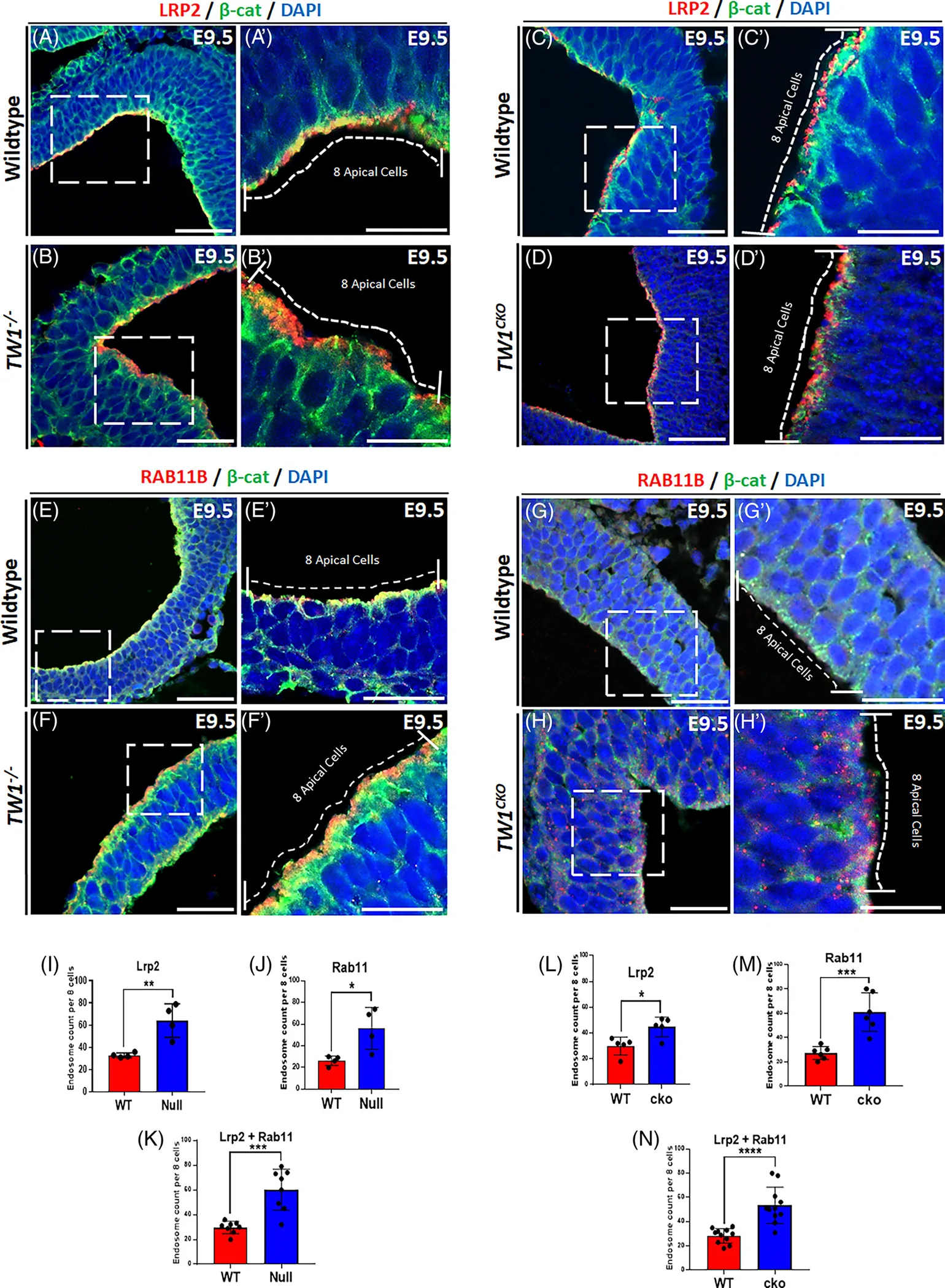
Expression pattern and quantification of endocytic vesicles in neuroectodermal cells. (A–B′) Immunofluorescence (IF) staining of LRP2 and β-catenin in E9.5 *Twist1* null and wild-type (WT) littermates. The endocytic vesicles were quantified in eight apical cells where β-catenin was used as a cell membrane marker to identify cell peripheries. (C, D) IF staining of LRP2 and β-catenin in E9.5 *Twist1* cko and WT littermates. (E–F′) IF staining of RAB11B and β-catenin in E9.5 *Twist1* null and WT littermates. (G–H′) IF staining of RAB11B and β-catenin in E9.5 *Twist1* cko and WT littermates. (I–K) The number of endocytic vesicles was increased in *Twist1* null using LRP2 and RAB11B and also in the combined quantification of both markers. (L–N) The number of endocytic vesicles was increased in *Twist1* cko using LRP2 and RAB11B and also in the combined quantification of both markers. For statistical analysis, the * represents a *p*-value <.05, ** represents a *p*-value <.01, *** represents a *p*-value <.001, and ****represents a *p*-value <.0001.

To determine the expression pattern of endocytic vesicle markers, dual IF staining for LRP2 and β-catenin as well as RAB11b and β-catenin was performed on WT *Twist1* null, and cko embryos at E9.5 and E10.5 (Figures 5 and S1). The IF images show that RAB11B and LRP2 are highly expressed in apical cells in both cko and null neuroectoderm when compared to corresponding WT tissues (Figure 5A–H′). However, the cells beneath the dorsal apical cells did not show robust expression of LRP2 and RAB11B, particularly in WT (Figure 5A′,C′,E′,G′). The increase in staining in mutant embryos suggests an elevation in the number of apical endosomes in the absence of *Twist1*. To investigate the level of change further, we counted the number of apical endocytic vesicles as a measure of quantitative analysis for endosome dynamics.

### 2.6 | Impact of *Twist1* on the Endocytic Vesicle Dynamic

To analyze changes in the number of endocytic vesicles, we counted the number of endocytic vesicles in eight apical neuroectodermal cells of IF images using the two markers, LRP2 and RAB11B. We used β-catenin as a cell-membrane associated marker to determine the cell peripheries. IF images of *Twist1* null and cko embryos at time points E9.5 and E10.5 depict a considerable increase in endosomal markers LRP2 (Figures 5A–D′ and S1) and RAB11B compared to WT (Figure 5E–H′). Most of the endocytic vesicles were found at the apical surface of the dorsal neuroectodermal cells in the WT, while for the null and cko, they were moderately shifted more towards the apical lateral regions. When we counted the apical endosomes for LRP2 in eight apical cells, we found that the number of endosomes increased around two-fold in *Twist1* null and cko compared to WT littermates of sections of the hindbrain (Figure 5I,L). A similar trend was seen in both *Twist1* null and cko for RAB11B (Figure 5J,M). When combined the quantification for both biomarkers, the LRP2 and RAB11B, *Twist1* null and cko showed a significant increase in apical endosomes which is consistent with the staining in individual IF images (Figure 5K,N). Our data demonstrate that the loss of TWIST1 significantly increases the number of endocytic vesicles at the apical surface of neuroectoderm during CNCC formation.

### 2.7 | mRNA expression of other endocytic regulator proteins

**FIGURE 6:**
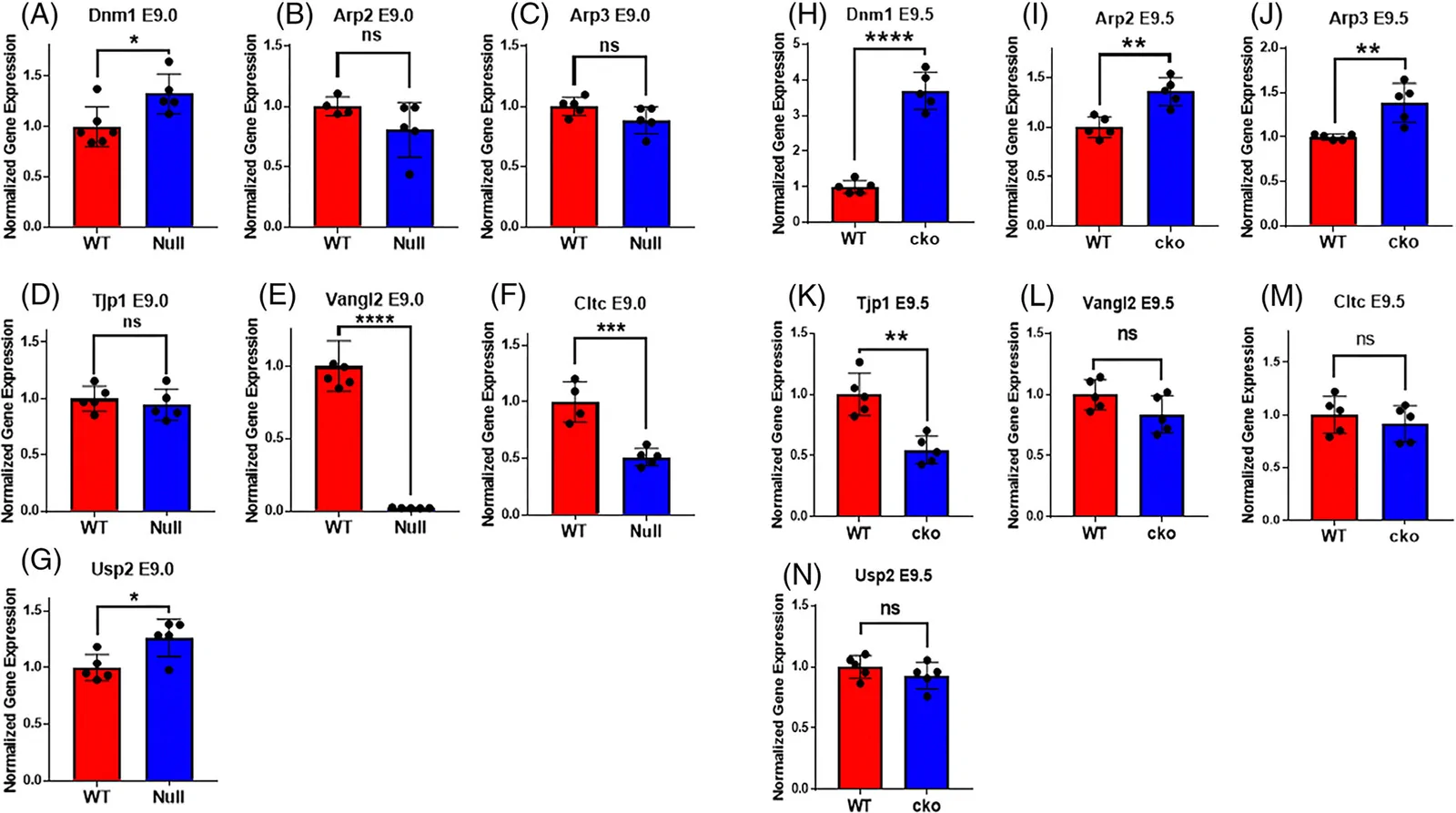
mRNA quantification of endocytic vesicle markers and associated genes. (A–G) A significant increase in the expression level of *Dnm1* and *USP2* was detected while the expression of *Vangl2* and *Cltc* was significantly decreased in E9.0 *Twist1* null compared to wild-type (WT) littermates. There was no significant change detected in remaining tested genes between mutant and WT embryos. (H–N) A significant increase in the expression level of *Dnm1*, *Arp2*, and *Arp3* was detected while the expression of *Tjp1* was significantly decreased in E9.0 *Twist1* cko compared to WT littermates. There was no significant change detected in remaining tested genes between mutant and WT embryos. For statistical analysis, the * represents a *p*-value <.05, ** represents a *p*-value <.01, *** represents a *p*-value <.001, and ****represents a *p*-value <.0001.

Apart from *Lrp2* and *Rab11b*, other endosomal genes involved in endosomal formation and recycling were also evaluated in *Twist1* null at E9.0 and *Twist1* cko at E9.5. We extracted RNA from hindbrain and first pharyngeal arch of WT and mutant embryos for quantification. We used three biological replicates for either WT and mutant and five technical replicants for each genotype for the real-time quantitative polymerase chain reaction assay. In *Twist1* null embryos, the expression of Dynamin1 (*Dnm1*) and Ubiquitin-specific peptidase 2 (*Usp2*) was significantly higher compared to WT littermates at E9.0 (Figure 6A,G). No statistically significant difference was found in the expression of Actin-Related Proteins 2 and 3 (*Arp2* and *Arp3*) and *Tjp1* between *Twist1* null embryos and WT (Figure 6B–D). However, we observed a decrease in the expression of VANGL Planar Cell Polarity Protein 2 (*Vangl2*) and Clathrin (*Cltc*) in *Twist1* null compared to WT samples (Figure 6E,F). When compared to WT, the level of *Dnm1* expression increased more than threefold in *Twist1* cko (Figure 6H). *Arp2* and *Arp3* showed a significant increase, whereas *Tjp1* expression showed a nearly 50% reduction in *Twist1* cko (Figure 6I–K). However, the expression levels of *Vangl2*, *Cltc*, and *Usp2* remain similar in WT and *Twist1* cko samples (Figure 6L–N).

### 2.8 | Quantification of the cell–cell interface in *Twist1* null and cko embryos

**FIGURE 7:**
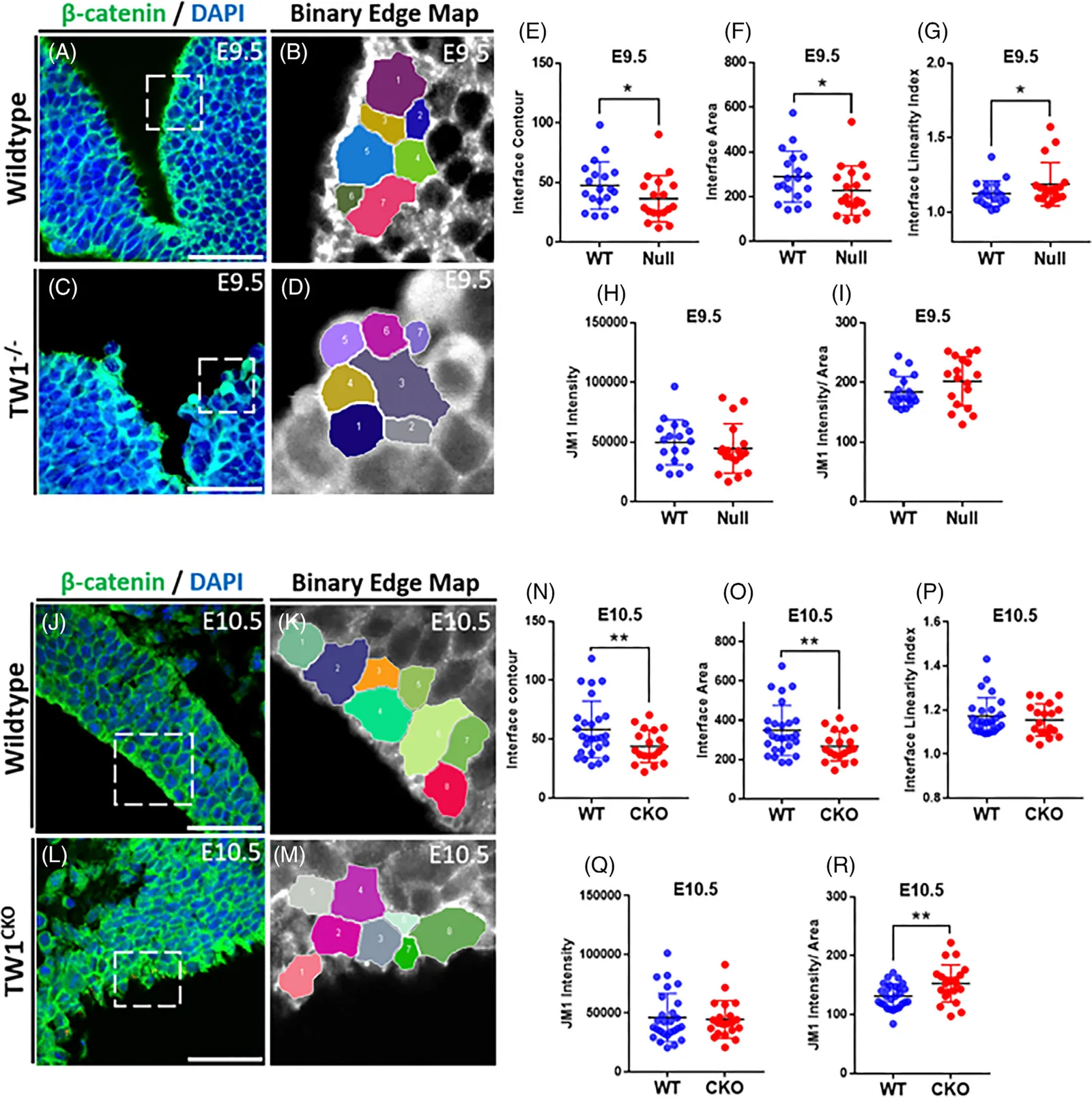
Quantification analysis of cell–cell junction of neuroectodermal cells of *Twist1* mutants and wild-type (WT) embryos. (A) Immunofluorescence (IF) staining of E9.5 WT mouse embryo using β-catenin as cell membrane marker. (B) Binary edge map of E9.5 WT mouse embryo of the cells used for quantification analysis. (C) IF staining of E9.5 *Twist1* null mouse embryo using β-catenin as cell membrane marker. (D) Binary edge map of E9.5 *Twist1* null mouse embryo of the cells used for quantification analysis. (E–I) Quantification analysis shows a decrease in the interface contour and interface area while there is an increase in the interface linearity index in *Twist1* null compared to WT. (H, I) There is no significant change detected in JM1 intensity and JM1 intensity/area between the mutant and WT embryos. (J) IF staining of E10.5 WT mouse embryo using β-catenin as cell membrane marker. (K) Binary edge map of E10.5 WT mouse embryo of the cells used for quantification analysis. (L) IF staining of E10.5 *Twist1* cko mouse embryo using β-catenin as cell membrane marker. (M) Binary edge map of E10.5 *Twist1* cko mouse embryo of the cells used for quantification analysis. (N–R) Quantification analysis shows a significant decrease in the interface contour and interface area while there is an increase in JM1 intensity/area in *Twist1* cko compared to WT. There is no significant change detected in interface linearity index and JM1 intensity between the mutant and WT embryos. Six biological replicates were used for IF staining and around 200 technical replicates were used for the cell–cell contact data analysis. For statistical analysis, the * represents a *p*-value <.05, ** represents a *p*-value <.01, *** represents a *p*-value <.001, and ****represents a *p*-value <.0001.

To determine the alternations in cell morphology of neuroectoderm during NT development, IF images for β-catenin at multiple time points were analyzed using the Junction Mapper software. Cell–cell interfaces for neuroectodermal cells near the apical side of the neural folds were analyzed based on interface contour, interface linearity index, interface area, junction marker intensity, and junction marker intensity per area. Images at the edges of the neural folds were chosen for *Twist1*^−/−^ with additional images included in the Supporting Information (Figures 7A–D and S2–S4). A significant decrease in interface contour and interface area was observed in *Twist1*^−/−^ compared to the WT (Figure 7E,F). However, there was a significant increase in the interface linearity index between *Twist1*^−/−^ and WT, and no significance in JM1 intensity and JM1 intensity per area (Figure 7G–I). Images at the edges of the neural folds were also selected for *Twist1*^*cko/−*^ with the corresponding WT (Figure 7J–M). A significant decrease in interface contour and interface area was observed for *Twist1*^*cko/−*^ compared to the WT (Figure 7N,O). There was no significant difference in the interface linearity index and JM1 intensity (Figure 7P,Q), while there was a significant increase in JM1 intensity per area of *Twist1*^*cko/−*^ compared to WT (Figure 7R). Altogether, a working model is proposed to illustrate how TWIST1 interacts with ciliary and endocytic vesicle factors that are involved in the cellular morphogenesis and actin cytoskeleton activity (Figure S5).

## 3 | DISCUSSION

Embryonic development is orchestrated through complex molecular and cellular processes governed by intricate interactions between genetic determinants, environmental influences, and coordinated signaling networks that direct cell lineage specification and differentiation.^37^ The multifaceted regulatory interactions within embryonic cells, combined with our incomplete mechanistic understanding, present significant challenges in elucidating the causative factors of craniofacial abnormalities, which represent the second most prevalent category of congenital birth defects.^16–21,24^ Identifying the regulatory pathways and associated cellular changes that drive tissue morphogenesis is therefore crucial for addressing fundamental biological concepts in developmental processes and the molecular basis of craniofacial disorders.

During neurulation, mesodermal signals from notochord induce the overlying dorsal ectoderm to differentiate into neural and non-neural ectoderm, establishing specified cell populations at the neural plate borders through tightly regulated gene expression programs essential for cell fate determination.^3,37^ Subsequently, the NT develops into the entire central and peripheral nervous system.^38,39^ This study aimed to elucidate TWIST1-dependent molecular and cellular alterations in neuroectoderm and determine why *Twist1* deficiency causes neuroectodermal disorganization and multiple ectopic lateral bending points along the dorsal neural folds leading to NT closure defects and brain abnormalities. We investigated whether TWIST1 colocalizes with adherens junction and endosomal proteins in neuroectodermal cells and whether *Twist1* loss alters primary ciliary length, endosomal marker expression, β-catenin levels, endocytic vesicle dynamics, and cell junction organization in neuroectodermal cells at dorsolateral regions. Our goal was to uncover unknown molecular and cellular alterations associated with NT defects in *Twist1* mutant mouse models. While our current data do not establish the precise mechanism by which TWIST1 impacts endocytic vesicle dynamics and ciliogenesis, they further our previous findings that demonstrated that TWIST1 protein interacts with cytosolic β- and δ-catenins as part of the adherens junction protein complex which affects cell shape changes and membrane activities.^12^

Recent transcriptomic analyses and quantitative in situ hybridization studies have demonstrated TWIST1 expression in neural plate borders and neural folds,^13,40^ providing compelling evidence for its direct functional role in neuroectodermal cellular development. Our immunostaining validated moderate TWIST1 expression in neuroectodermal cells during NT development. *Twist1* null and conditional knockout mouse models exhibit failed cranial NT closure, formation of multiple ectopic bending points, and disrupted CNCC fate transition and survival, resulting in significant craniofacial bone deficiencies.^12^ To determine *Twist1’s* impact on primary ciliogenesis, we examined cilia length and number using the ciliary membrane marker ARL13B. Our data revealed significantly reduced ciliary length at the dorsal *Twist1* null neuroectoderm compared to control littermates at E9–9.5. The number of ciliated cells also appears to be reduced in mutant neuroectodermal cells. Alterations in endocytic vesicle protein expression and β-catenin levels, along with increased endocytic vesicle abundance, could potentially explain the shortened cilia in mutant embryos. These findings suggest that TWIST1 is involved in adherens junction integrity and endocytic vesicle dynamics, which could explain the changes observed in cell–cell junction and intercellular spatial organization. These cellular processes facilitate apical constriction, membrane-associated protein recycling, and cell fate transitions during neurulation.^12,41^

In *Twist1’s* absence, neuroectodermal disorganization accompanied by multiple bending points and shorter cilia may help explain NT closure defects in *Twist1* null embryos and exencephaly in *Twist1* conditional knockouts.^12^ The altered expression of genes involved in endocytic vesicle function and elevated endocytic vesicle numbers are consistent with observed increase in LRP2 and RAB11B proteins in *Twist1* null and conditional knockout hindbrain embryos. Our previous and current findings demonstrate *Twist1’s* integral role in NT closure and cell fate transition of pre-migratory CNCC^12^. We demonstrate that TWIST1 expression is required for proper neural fold bending and cell–cell junctions in regions where pre-migratory CNCCs delaminate from neural fold edges, and that loss of *Twist1* leads to ectopic bending points. It is important to emphasize that apical constriction across the neuroectoderm is essential for proper neural fold bending; even moderate levels of *Twist1* expression in these cells could be crucial for cellular changes. Moreover, it has been reported that some developmental regulators exert potent effects even when expressed at relatively low levels.^42,43^ The significance of these molecular and cellular findings is underscored by the current absence of direct evidence linking TWIST1 dysfunction to cranial NT closure defects in human cases, despite the fact that the etiological basis for most human NT defects, including anencephaly and exencephaly, remains largely undefined.^44^ Based on our findings, we suggest a link between *TWIST1* locus and downstream target genes involved in NT development. Interestingly, recent examination of *TWIST1* regulatory elements led to the identification of mutations linked to Auriculocondylar syndrome^45^ and limb malformations.^46^ These investigations may reveal previously unrecognized connections between TWIST1 dysfunction and birth defects associated with NT disorders, potentially opening avenues for diagnostic and therapeutic interventions.

## 4 | EXPERIMENTAL PROCEDURE

### 4.1 | Mouse Strains

All mice used in this study were generated using a C57BL/6J mouse genetic background. *EIIA-Cre* mouse line was crossed to the B6;129S7-*Twist1*^*fl/fl*^ mice to obtain the *Twist1* heterogeneous mice, as previously described.^12^ *Twist1* cko in neuroectoderm was generated using the *Wnt1-Cre2* deleter strain. The number of experimental animals was calculated based on the power analysis. The animal work was approved by the CLAMC committee at UTHealth Houston under the Approved Animal protocol number AWC-22–0060.

### 4.2 | Histological and Immunofluorescent Staining

Embryonic tissues from control, *Twist1*^−/−^, and *Twist1*^*cko/−*^ mice at E9–9.5 were embedded in paraffin, sectioned, and stained with hematoxylin and eosin (H&E) for histological analysis. For IF staining, homozygous *Twist1 null* and cko mutant embryos at E9–9.5 were identified via phenotyping of the NTs, either open or malformed neural folds. Littermate embryos with phenotypically normal NTs were used as control. The WT *Twist1*^+*/*+^, *Twist1*^*cko/−*^, and *Twist1*^−/−^ embryos at later timepoints were genotyped via PCR as described previously.^47^ Embryos were collected for detecting the expression pattern and level of endocytic markers and adherens junction proteins. Expression of LRP2, RAB11b, and β-catenin was performed on head sections of paraffin-embedded mouse embryos. The immunofluorescent staining protocol was followed as previously described.^47^ The primary antibodies used for the staining are monoclonal TWIST1 (1:150, Abcam, MA), mouse monoclonal anti-β-catenin (1:200, Santa Cruz), rabbit monoclonal anti-RAB11B (1:130, ABclonal), and rabbit monoclonal anti-LRP2 (1:150, ABclonal). The sections were incubated for 3 h with secondary antibodies conjugated to Alexa fluorophores 488 goat anti-rabbit (1:150) and 555 goat anti-mouse (1:150). The sections were counterstained for nuclei with water-diluted DAPI. The mounted sections were imaged using a Nikon C2 Confocal microscope.

### 4.3 | Cilia Staining and Measurements

E9.0–E9.5 embryo sections were used for cilia staining. Antigen retrieval was performed by heating slides in sodium citrate buffer (10 mM sodium citrate, 0.05% Tween 20, pH 6.0) at 96°C for 10 min. Slides were washed in PBS, permeabilized using 0.5% Triton X-100 in 1× PBS for 30 min, washed in PBS again, then blocked in 10% normal goat serum. Primary and secondary antibodies were incubated following the same method as described above. Primary antibody for ARL13B (1:300, Proteintech, 17711–1-AP) and secondary antibody of Goat anti-Rabbit IgG (H + L) Alexa 594 (1:500 tissue Invitrogen; A-11037) were used. DAPI (5 μM) was used to mark nuclei. Images were acquired using the Nikon CSU-W1 Spinning-disk confocal 60x objective and an intermediate 4x magnification changer with SoRa module. A z-stack was captured with slices taken every 0.2 μm ensuring the entire tissue section was captured. A max intensity projection (MIP) was created for measurement. Cilia lengths were measured using ImageJ software and a segmented line was used to trace the cilium.^48,49^ The length in pixels was converted to microns using the pixel conversion factor for the objective used. MIP image was used to count the % ciliation in the neuroectoderm.

### 4.4 | RT-qPCR

The mRNA expression of *Lrp2*, *Rab11b*, and *β-catenin* in the mid-and hindbrain regions of mouse embryos was measured in three pooled biological and five technical replicates at E8.5 and E9.0 for *Twist1* null embryos, and E9.5 and E10 for *Twist1* cko compared to WT littermates. We also quantified the expression of genes involved in endocytosis, including *Vangl2*, *Cltc*, *Dnm1*, *Tjp1*, *Usp2*, *Arp2*, and *Arp3*.

### 4.5 | Western blot assay

Midbrain and hindbrain tissues were dissected from embryos at E9.5, E10, and E10.5 and used to extract total protein. Tissues were directly frozen and thawed twice after grinding tissues with a plastic pestle on dry ice. The samples were sonicated for 15 s at 35 power level, then centrifuged at 14,000 rpm for 10 min at 4°C to remove undissolved substances. To denature the proteins, the lysates were boiled for 10 min with 1× Laemmli buffer, loaded into 4–20% gradient sodium dodecyl sulfate–polyacrylamide gel electrophoresis, and the protein amounts were analyzed as described previously.^47^ We used primary antibody for RAB11B, β-catenin, and GAPDH incubated overnight in 3.5% bovine serum albumin and secondary antibody conjugated to HRP for detecting protein levels.

### 4.6 | Endosome quantification

We measured the number of apical endosomes in *Twist1* mutant and WT embryos to determine the impact of *Twist1* deletion on endocytic vesicle markers, LRP2 and RAB11B, and the number of endosomes. Immunofluorescent images of 3 different biological samples were collected for WT, *Twist1* cko, and null embryos. Dual immunofluorescent staining for the endocytic factors LRP2 and β-catenin, as well as RAB11B and β-catenin, was performed to determine the location and number of the endocytic vesicles along the apical cell membrane. Using a multi-point tool in the software Image J^1^ version 5.3k, we manually counted the vesicles with red signals of eight apical cells at either the dorsolateral hinge points and near midline in neural folds. A distinctly visible red fluorescent vesicle or yellow when overlaps with green β-catenin was counted as one vesicle, while for not so distinct red signal due to the densely packed endosomes, we avoided counting these vesicles unless we could estimate it as two vesicles depending upon the size covered compared to a single vesicle.

### 4.7 | Quantification of cell–cell junctions

Junction mapper software was used to quantify cell–cell junctions for apical cells at neural fold edges where pre-migratory CNCC usually emerged and delaminated.^50^ Cell–cell junctions were compared between WT and *Twist1* null and cko samples at E9.5 and E10.5. β-Catenin was used as the junction marker for quantification due to prominent expression at the cell membrane. Interface contour, interface linearity index of neuroectodermal cells, interface area, and junction marker intensity per area were measured and compared with WT. Eight apical cells were picked within similar regions for comparison. Quantification was performed for cell–cell junctions of cells at the neural fold edges. Four cells directly along the apical membrane were chosen, along with the four cells directly behind them, for a total of eight cells per region. Another method of choosing cells to quantify involved choosing eight cells directly along the apical membrane that were lined up in a linear manner. However, this method proved more difficult due to gaps and inconsistent spacing along the membrane, which led to more significant variation among the cells.

### 4.8 | Statistical analysis

All quantitative data are presented as the means ± standard deviations. Statistical comparisons were conducted using the unpaired student’s *t*-test for two groups, WT and mutant, using GraphPad Prism 8 software. A significance level of *p*-value <.05 was considered statistically significant.

## Supplementary Material

Suppl.Figure 1

Suppl.Figure 2

Suppl.Figure 3

Suppl.Figure 4

Suppl.Figure 5

Additional supporting information can be found online in the Supporting Information section at the end of this article.

## Data Availability

All data can be found within the article and its Supporting Information. Additional inquiries can be directed to the authors of this study.
