# Supplementary figures and images for: Loss of Twist1 leads to disruption of ciliary length, endocytic vesicle dynamics, and cell–cell junctions during neural tube formation
Source: Dev Dyn. Author manuscript; Available in PMC 2026 May 8. (PMC13154908; doi:10.1002/dvdy.70109)

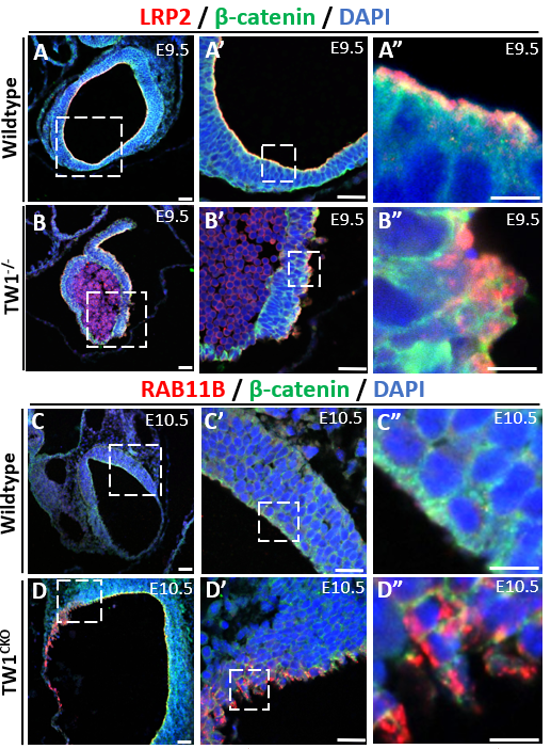

Supplement: Suppl.Figure 1 [file NIHMS2143106-supplement-Suppl_Figure_1.png]

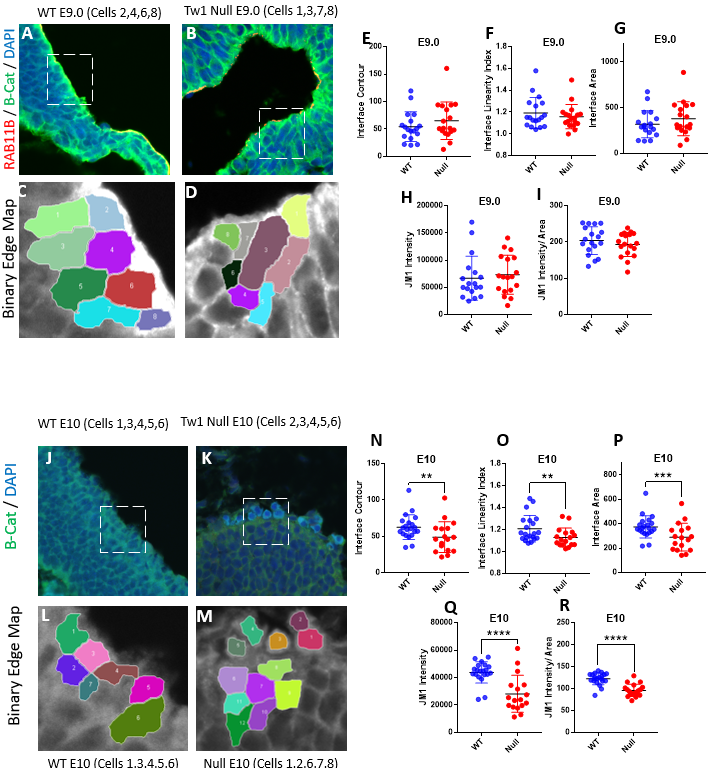

Supplement: Suppl.Figure 2 [file NIHMS2143106-supplement-Suppl_Figure_2.png]

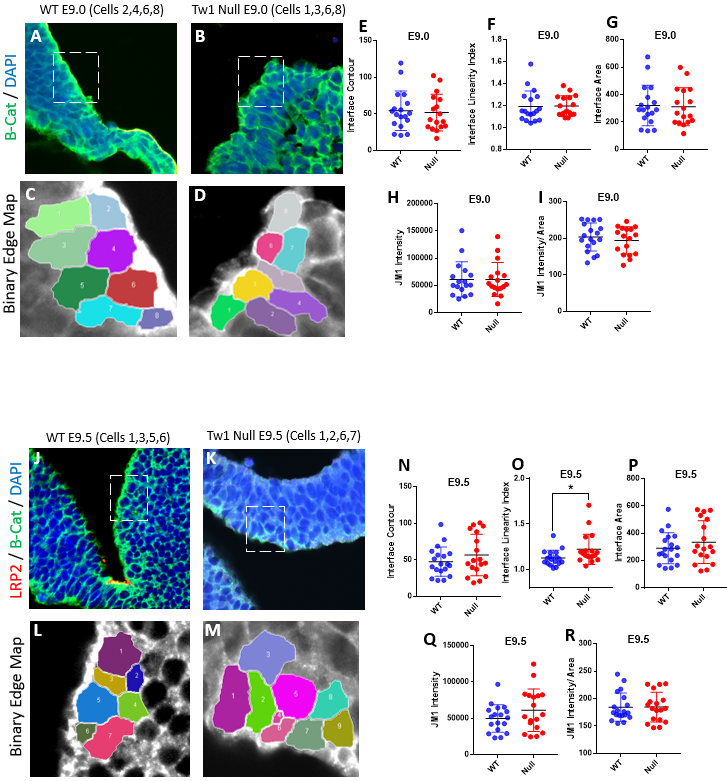

Supplement: Suppl.Figure 3 [file NIHMS2143106-supplement-Suppl_Figure_3.png]

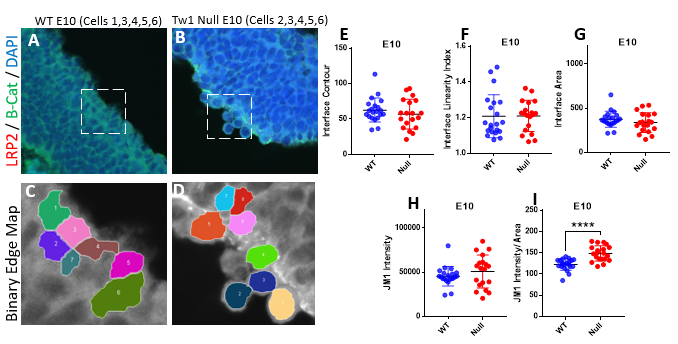

Supplement: Suppl.Figure 4 [file NIHMS2143106-supplement-Suppl_Figure_4.png]

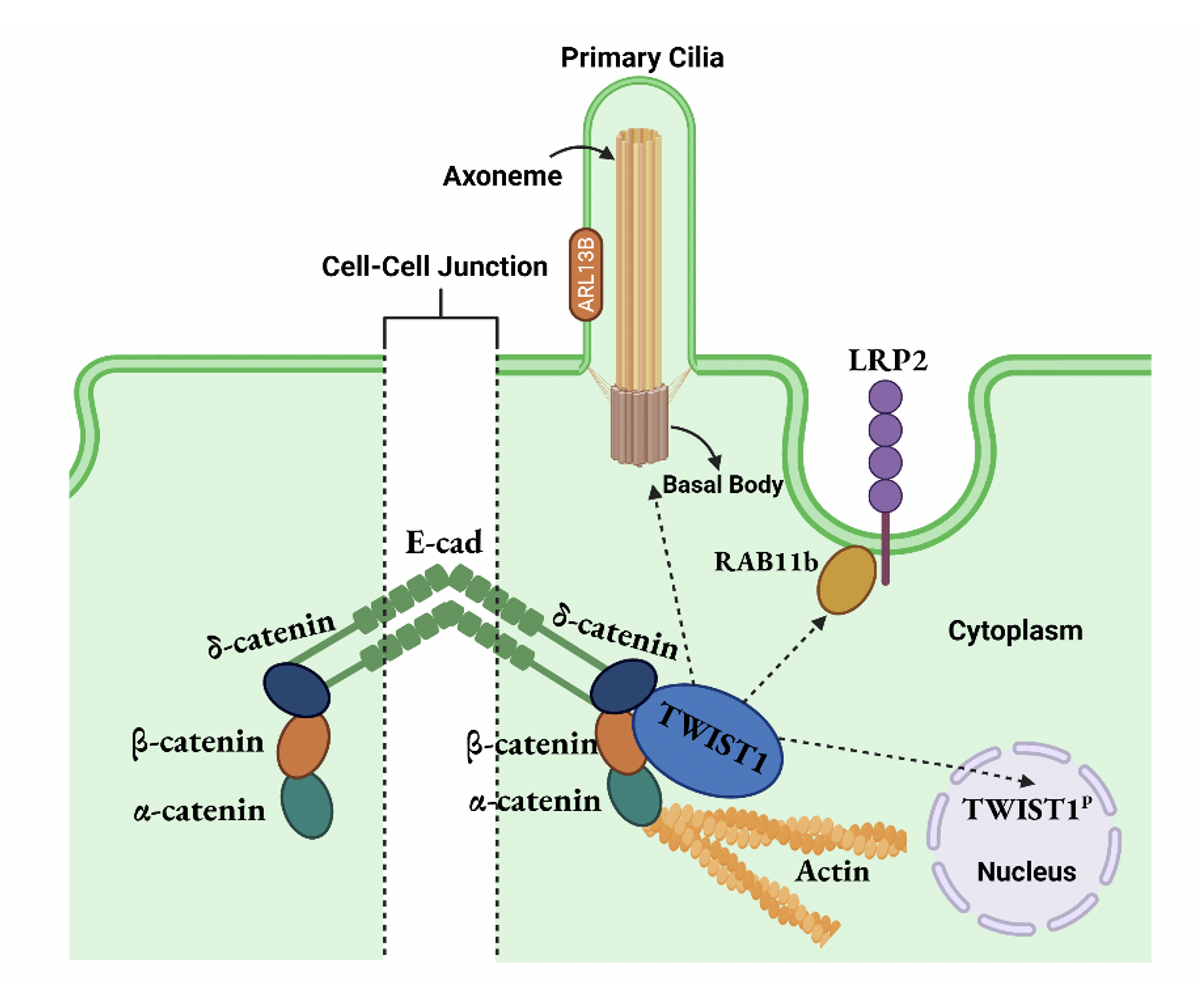

Supplement: Suppl.Figure 5 [file NIHMS2143106-supplement-Suppl_Figure_5.png]
